# Using Artificial Neural Network Modeling to Analyze the Thermal Protective and Thermo-Physiological Comfort Performance of Textile Fabrics Used in Oilfield Workers’ Clothing

**DOI:** 10.3390/ijerph18136991

**Published:** 2021-06-30

**Authors:** Sumit Mandal, Nur-Us-Shafa Mazumder, Robert J. Agnew, Indu Bala Grover, Guowen Song, Rui Li

**Affiliations:** 1 Department of Design, Housing and Merchandising, Oklahoma State University, Stillwater, OK 74078-5061, USA; shafa.mazumder@okstate.edu; 2 Fire Protection and Safety Engineering and Technology Program, Oklahoma State University, Stillwater, OK 74078-5061, USA; rob.agnew@okstate.edu; 3 Department of Computer Engineering, YMCA Institute of Engineering, Faridabad 121006, India; indu.mtech.ymca@gmail.com; 4 Department of Apparel, Events, and Hospitality Management, Iowa State University, Ames, IA 50011-2100, USA; gwsong@iastate.edu (G.S.); ruili@iastate.edu (R.L.)

**Keywords:** oilfield workers’ clothing, protective textiles, sweat moisture, microclimate air gap, thermal protective performance, thermo-physiological comfort performance

## Abstract

Most of the fatalities and injuries of oilfield workers result from inadequate protection and comfort by their clothing under various work hazards and ambient environments. Both the thermal protective performance and thermo-physiological comfort performance of textile fabrics used in clothing significantly contribute to the mitigation of workers’ skin burns and heat-stress-related deaths. This study aimed to apply the ANN modeling approach to analyze clothing performance considering the wearers’ sweat moisture and the microclimate air gap that is generated in between their body and clothing. Firstly, thermal protective and thermo-physiological comfort performance of fire protective textiles used in oilfield workers’ clothing were characterized. Different fabric properties (e.g., thickness, weight, fabric count), thermal protective performance, and thermo-physiological comfort performance were measured. The key fabric property that affects thermal protective and thermo-physiological performance was identified as thickness by statistical analysis. The ANN modeling approach could be successfully implemented to analyze the performance of fabrics in order to predict the performance more conveniently based on the fabric properties. It is expected that the developed models could inform on-duty oilfield workers about protective and thermo-physiological comfort performance and provide them with occupational health and safety.

## 1. Introduction

According to the U.S. Department of Labor statistics, 1566 workers died from injuries in the oil-and-gas drilling industry and related fields from 2008 through 2017 [1]. Additionally, a recent explosion in one of Oklahoma’s oil-and-gas rigs resulted in the deaths of five workers [2]. Particularly, Oklahoma is among the 10 most wildfire-prone states in the USA (Insurance Information Institute, New York, NY, USA, 2018 statistics). Notably, the majority of these fatalities and burn injuries resulted from inadequate protection and comfort provided by oilfield workers’ thermal protective clothing [3,4].

The thermal environment where the on-duty oilfield workers work most often decides the thermal protective performance of their fire protective textiles [5]. The thermal environments faced by these workers have been investigated by many researchers to understand the performance of fire protective textiles [5,6,7,8,9]. These investigations have found that oilfield workers in the field are exposed to radiant heat, flames, hot surfaces, steam, and hot liquids, which can be of various intensities and for different durations. The characteristics of the fabrics used in fire protective textiles mostly decide the performance of the workwear under thermal exposure. The performance of the fabrics used to make oilfield workers’ workwear needs to be studied and understood under different thermal exposures in order to improve the protection performance. Moreover, it has been found that the metabolic heat and sweat vapor from the wearers’ body to the ambient environment may not properly transfer through the fabrics used in the workwear. This eventually will cause significant heat stress and strain on their bodies. Therefore, thermo-physiological comfort performance of the fabrics used in workwear also needs to be studied and understood.

Both the thermal protective performance and thermo-physiological comfort performance of fabrics used in protective clothing contribute significantly to reducing the number of oilfield workers’ skin burns and heat stress [3,6,10]. Various standardized test methods by ASTM (American Society for Testing Materials) or ISO (International Organization for Standardization) were used under different thermal exposure to measure the protective and comfort performance of the fabrics [11,12,13,14,15]. However, these standardized laboratory testing techniques are fabric destructive in nature, time consuming, and/or expensive to carry out on a regular basis [16,17]. Considering this, previous studies have focused on developing empirical models to predict the protective and comfort performance of the fabrics used to make workwear based on their physical properties [18,19,20]. For developing the models, the key fabric properties (e.g., thickness, thermal resistance, evaporative resistance) were identified first, which affect the protective and comfort performance of the workwear. Secondly, multiple linear regression (MLR) and/or artificial neural network (ANN) models were developed by employing the identified key fabric properties to conveniently predict their performance. For example, a group of researchers [21,22,23,24,25] found that weight, thickness, and thermal and evaporative resistance are the key fabric properties that affect the fabric’s thermal protective performance. On the other hand, these researchers concluded that the thermo-physiological comfort performance is mostly affected by fabric weight, evaporative resistance, and water spreading properties. In most of these research papers, it was found that ANN models more accurately predicted the performance than did MLR models [20,26]. Notably, Udayraj et al. (2017) found that ANN model predictions agreed better with the experimental observations [27]. After comparing the prediction performance of both ANN and MLR models, Mandal et al. (2018) also have found that the ANN model gives more accurate predictions compared to those of the MLR model [25]. This is because the ANN models are more advanced statistical learning models in the machine learning and cognitive science disciplines. They are inspired by biological neural networks and are used to estimate or approximate a variable that is dependent on a large number of input variables. As the protective and comfort performance is dependent on a large number of fabric properties, ANN models performed better in comparison to the MLR models in the previous studies.

Although previous studies recommended using ANN models to predict fabrics’ performance, these models were developed based on the experimental thermal protective performance values of dry fabrics only. As oilfield workers’ sweat profusely while firefighting, this sweat moisture could affect the thermal protective performance of fabrics [28,29]. Additionally, previous studies were carried out while developing the ANN models without considering the air gap that results in microclimates between the fabrics and the wearers’ (oilfield workers’) bodies. Contextually, many researchers identified that absorbed moisture plays a crucial and complicated role in the performance of fire protective textiles since the thermal conductivity and heat capacity of the water is higher than that of the fabric and air. The significance of the effect on the performance depends on the amount and location of the moisture in the clothing [28,30,31,32,33,34,35,36,37]. Moreover, the entrapped air gap has great positive influence on the protective performance of fire-protective textiles but has a negative effect on comfort performance [38,39,40,41,42,43,44,45,46,47,48]. In this context, Deng et al. (2018) found that air gaps within the clothing layer and human skin play an important role in determining the thermal protective and comfort performance, and this air gap should be considered as a parameter while developing a performance-predictive model [38]. Therefore, to reproduce more realistic conditions, moisture and the microclimate air gap have to be considered as they can substantially influence the thermal protective and thermo-physiological comfort performance of fabrics [29]. Thus, an extension of the modeling studies on protective and comfort performance is needed that considers moistened fabrics and the microclimate air gap.

This research aimed to develop sophisticated computational models in order to fulfil the above-mentioned knowledge gaps. In this work, the ANN modeling approach was used for a more realistic prediction of thermal protective and thermo-physiological comfort performance of fabrics by considering sweat moisture and the microclimate air gap. For this, we characterized the thermal protective and thermo-physiological comfort performance of fabrics in order to identify the key fabric properties affecting performance. By employing the key fabric properties, an ANN model was developed for predicting performance. Notably, the developed model could lead to prediction of the thermal protective performance of fabrics under flame and radiant heat exposure. 

## 2. Materials and Methods

**Figure 1:**
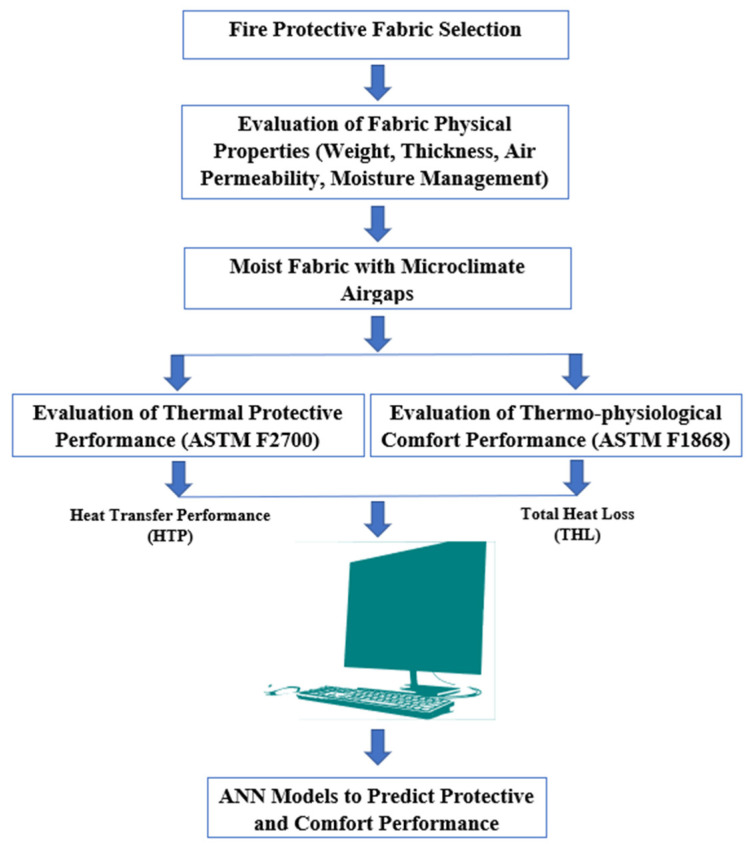
Research methodology.

For this research (Figure 1), different commercially available fabrics used in oilfield workers’ protective clothing were selected and their physical properties including fabric count (number of ends and picks (i.e., longitudinal warp and weft threads) within the fabric) were measured using the standardized ASTM test methods (Table 1). The thermal protective and thermo-physiological comfort performance of the fabrics with different levels of moisture and a microclimate air gap size were determined by using ASTM F 2700 and ASTM F 1868 standards, respectively. Here, the protective performance and comfort performance of the fabrics were measured in terms of heat transfer performance (HTP) and total heat loss (THL), respectively, and systematically tabulated and presented. The changes in HTP and THL with different levels of moisture and air gap are also statistically discussed. The statistical significance tests and 95% confidence interval (CI) tests were carried out to identify the difference in HTP/THL under different levels of moisture and air gaps; in this study, if the *p*-value of mean difference in HTP/THL at two different conditions of moisture or air gap was less than 0.05, the difference was interpreted as a significant difference. Based on the sign (+ or −) of the upper and lower levels of HTP/THL in the 95% CI tests, whether the difference in HTP/THL was significantly higher or lower was interpreted. Descriptive statistical analysis was carried out to understand the impact of different selected fabrics on the HTP and THL. The added water and air gaps also were considered as ordinal parameters during the descriptive statistical analysis. The findings in the difference of HTP and THL were justified based on the theory of textile science as well as heat and mass transfer. 

### 2.1. Thermal Protective Performance Test

**Figure 2:**
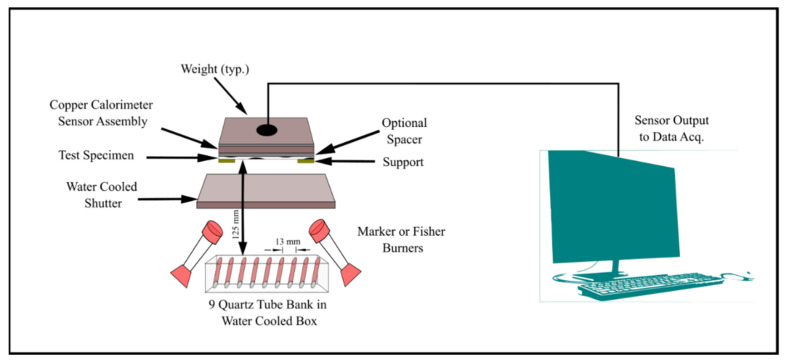
Thermal protective performance tester.

The thermal protective performance of the fabrics was measured under 2 ± 0.05 cal/cm^2^ s heat flux with a combination of convective and radiant heat exposure using the ASTM F 2700 standard as shown in Figure 2 [16]. The samples were positioned horizontally and the unsteady-state heat transfer through the specimen was measured using a copper slug calorimeter. The transmitted heat sensor was a 4 ± 0.05 cm diameter circular copper slug calorimeter constructed from electric grade copper. This standard was designed to measure the heat transfer performance of materials that are exposed to combined convective and radiant thermal hazards. Maker or fisher burners, two of each, with a diameter of 38 mm top and 1.2 mm orifice size were used as one of two thermal energy sources. As the second source, nine 500 W T3 translucent quartz infrared lamps were arranged in a liner array with 13 ± 0.5 mm spacing between center to center, and 125 ± 10 mm from the specimen surface. Testing was carried out on two different air gaps (0 and 6 mm) with three different amounts of moisture (0 (dry), 20, and 50%). Self-designed air gap sizes that are equivalent to the ASTM standards requirements were chosen in this study. Additionally, 0% moisture was the base line of measuring the protective performance; then, moisture was increased up to 50%. Previous studies showed that the protective performance is lowest at 20% added moisture and steadily increases up to 50% [30]. This led us to choose 0, 20, and 50% moisture in this study. The air gaps between the sensor and the fabric were created by using a 6 mm spacer between the sensor assembly and the back surface of the specimen. The required amount of distilled water was sprayed on the back side of the fabric surface to simulate the absorbed sweat. In this study, distilled water was used; human sweat can be either acidic or alkaline, and different solutions might have different effects on performance, so distilled water was used to keep the effects consistent. Three samples of 150 by 150 ± 5 mm were prepared for testing for each testing scenario. In total, there were six different testing scenarios for each fabric type. The average value of three samples was taken to determine the heat transfer performance value. Sample exposure was terminated when the total accumulated thermal energy measured by the calorimeter met the following empirical performance curve: cal/cm^2^ = 1.1991 × *t_i_*^0.2901^, where *t_i_* is the time since the initiation of the thermal exposure. This time value determines the heat transfer performance (HTP) value for test specimen and is given by J/cm^2^ or cal/cm^2^. The fabric with a higher HTP value usually has higher resistance to flame and should provide more heat protection to the wearer. 

Heat transfer performance (HTP) value was calculated as cal/cm^2^ = *t_Intersect_* second × calibrated burner heat flux value, cal/cm^2^ s.

### 2.2. Thermo-Physiological Comfort Performance Test

**Figure 3:**
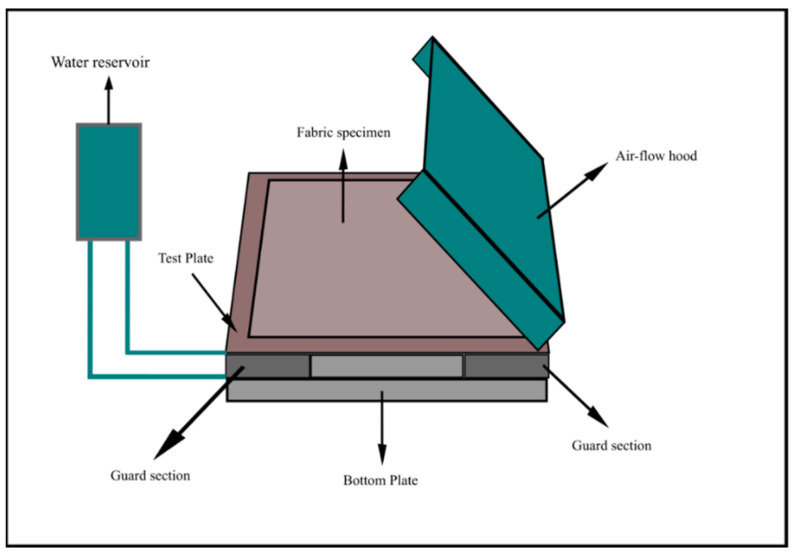
Thermo-physiological comfort performance tester.

The thermo-physiological comfort performance of the fabric was measure using the ASTM F 1868 standard test method (Figure 3). A guarded flat plate was composed of a test plate, guard section, and bottom plate, each electrically maintained at a constant temperature in the range of human skin temperature between 33 and 36 °C. The size of the samples was 450 × 450 mm and was large enough to cover the surface of the hot plate test section and the guard section completely. Testing was carried at 0 and 6 mm air gaps in both dry and wet conditions. In order to consistently compare the thermo-physiological comfort performance data set with the protective performance data set, this study chose 0 and 6 mm air gaps for the protective performance test. The air gap between the sensor and the fabric was created in a similar way to that used in the thermal protection performance test, by using a 6 mm spacer between the sensor assembly and the back surface of the specimen. There were four different testing scenarios for each fabric. In total, 12 samples (3 samples for each testing scenario) were prepared from each fabric type for testing. Values of three samples from each fabric were measured and averaged. Thermal resistance is the resistance to the flow of heat from a heated surface to a cooler environment. Total resistance to dry heat transfer (*R_ct_*) for a fabric was calculated using the following formula:

*R_ct_* = (*T_s_* − *T_a_*) × *A/H_c_*

where *R_ct_* is the total resistance to dry heat transfer provided by the fabric system and air layer (C.m^2^/W), *A* is the area of the plate test section (m^2^), *T_s_* is the surface temperature of the plate (°C), *T_a_* is the air temperature (°C), and *H_c_* is the power input (W). The value of *R_cf_*, which is the intrinsic thermal resistance of the fabric alone, is determined by subtracting thermal resistance the value measured for the air layer *R_cpb_* (bare plate test) from the average total thermal resistance value measured for the fabric system and air layer, *R_ct_*_._

Evaporative resistance is the resistance to the flow of moisture vapor from a saturated surface to an environment with a lower pressure. Total evaporative heat transfer resistance was calculated by using the following formula:

*R_et_* = (*P_s_* − *P_a_*) × *A/H_E_*

where *R_et_* is the total resistance to evaporative heat transfer provided by the fabric system and air layer (Pa·m^2^/W), *A* is the area of the plate test section (m^2^), *P_s_* is the water vapor pressure at the plate surface (Pa), *P_a_* is the water vapor pressure in the air (Pa), and *H_c_* is the power input (W). Averaging the apparent intrinsic evaporative resistance of all specimens (over the equilibrium period and a minimum of six samples) determines the average apparent intrinsic evaporative resistance $R_{ef}^{A}$ of the laboratory sample. 

Total heat loss (THL) of the sample was measure by using the following formula:

$$Q_{t}\;=\frac{10\;{}^{\circ}C\;\;}{R_{cf}+0.04}+\frac{3.57\;kPa}{R_{ef}^{A}+0.0035}$$

where *Q_t_* is the total heat loss (W/m^2^), *R_cf_* is the average intrinsic thermal resistance of the laboratory sample (K m^2^/W), and $R_{ef}^{A}$ is the average apparent intrinsic evaporative resistance of the sample (Pa·m^2^/W). The total heat loss (THL) value is given by W/m^2^ and is inversely proportional to the thermal and evaporative resistance. Higher THL values indicate higher heat transfer through the fabric, which is proportional to the comfort performance; however, THL is inversely proportional to the thermal protection performance. 

### 2.3. Characterization Procedure to Identify Key Fabric Properties

To identify the key fabric properties affecting the thermal protective (i.e., HTP) and thermo-physiological comfort (i.e., THL) performance of dry and moistened fabrics with and without an air gap (Figure 1), the linear regression between fabric properties and performance was analyzed using the SPSS software. In this study, two basic fabric properties, thickness and air permeability, were considered for the linear regression. These properties can be easily measured and they are mutually independent. As other fabric properties such as fabric count, weight, thermal resistance, and evaporative resistance are mutually dependent on thickness and/or air permeability, it has been hypothesized that the selected two fabric properties can properly represent the linear regression with performance. Different studies used linear regression analysis to find out the relation between fabric properties and performance [49,50]. For the statistical analysis, the mentioned fabric properties in Table 1 were considered as independent variables (input) and the amount of moisture and air gap size were the ordinal independent parameters. Two separate regression analyses were completed where dependent variables were HTP and THL, respectively. The statistical analyses were carried out at a 95% CI; therefore, *p*-values less than 0.05 were considered statistically significant. Among the thickness and air permeability properties, the properties that showed the highest absolute regression coefficient values were considered the key fabric properties affecting the performance. The impact of key fabric properties on the performance was justified in association with the scientific theory on heat and moisture transfer through porous fabrics (e.g., conduction, convection, evaporation). Altogether, it was interesting to understand how the key fabric properties affected the protective and comfort performance in more realistic conditions, i.e., sweat moisture and with microclimate air gaps. 

### 2.4. Procedure for ANN Modeling

Further, an ANN modeling technique was used to predict the protective and comfort performance of moistened fabrics with microclimate air gaps using the key fabric properties in MATLAB R2019a. Notably, ANN is an empirical approach that is widely applied to capture and represent any kind of relationship between the input (e.g., key fabric properties) and output (e.g., fabric performance) variables. In this study, different ANN models were constructed for predicting the protective performance of fabrics. An ANN model consists of at least three layers: input, hidden, and output layers. Each neuron in a layer has adjustable weights for its inputs and an adjustable bias. For constructing an ANN model, values for hyper-parameters, e.g., number of hidden layers and number of neurons in a single hidden layer, and choice of activation functions also need to be specified. This study employed a multilayer perceptron (MLP) architecture with three-layers, a feed-forward network with one hidden layer and a hyperbolic tangent sigmoid transfer function as an activation function in the hidden layer, and the linear function in the output layer. Next, the trial-and-error method was used to choose the optimum number of neurons in the hidden layer. For this, the feed-forward ANN models were trained using the supervised training form, the Levenberg–Marquardt optimization (trainlm) backpropagation method, with 1–10 neurons, and the best predictive ANN model was selected for deciding the number of neurons in the hidden layer. One of the potential problems with supervised learning is overfitting. In case of overfitting a model, it shows a very low value for error on the training set, but a very high value for test error or generalized error when new data is presented to the model. Generalization is a term that is used to explain how accurately a trained model can make predictions when presented with new data, i.e., we want the gap between the training and test error to be low. To improve the generalization and prevent overfitting of the ANN models, some regularization was needed. This was achieved indirectly by employing the most popular regularization technique of early stopping. For this, by default, MATLAB software randomly divided 70% of the data for the training, 15% of the data for the validation, and the remaining 15% of the data for testing the prediction performance of the ANN models. In this study, the performance of the ANN models were analyzed based on the Pearson correlation coefficient (r) and root mean square error (RMSE) of the models. In the early stopping method, the validation and training errors usually decrease in the early phases of training. However, when the model starts to overfit, the validation error starts increasing for a specified number of iterations, training is stopped at this time, and the ANN weights and biases at the minimum of the validation error are returned. 

## 3. Results and Discussion

The HTP and THL values of the selected fabrics (obtained by using the methods described in Section 2.1 and Section 2.2) are presented in Table 2. 

Table 2 shows that the HTP values of the fabrics increase with increasing moisture percentage in the fabrics. This is because the presence of moisture within the fabrics could enhance the heat capacity of the fabrics [51]. Due to the increase in heat capacity of the fabrics, a significant amount of thermal energy could be stored within the fabrics [52]. As a result, much less thermal energy could transfer through the fabrics to the wearers’ skin. This situation results in higher thermal protective performance of the fabrics. In fact, no heat transfer occurs in some fabrics such as A and B. Based on Table 1, it is clear that the air permeability values of fabrics A and B are much lower than those of fabrics C, D, and E. This means that fabrics A and B have a tighter and less porous structure in comparison to the that of fabrics C, D, and E. The absorbency rating of the fabrics also suggested similar results. Fabrics A and B did not absorb any water in the spray test and remained completely dry. There might be additional polymer finishing in fabrics A and B, which resulted in low water absorbency and air permeability. Due to this structural difference, moisture could be trapped on the surface and/or inside the fabrics and enhance the heat capacity of the fabrics. Due to this, the HTP values of these fabrics were very high. Notably, the HTP value of fabric B was higher than that of fabric A, even though both the fabrics comprised similar fibers and weight. This is because the thickness of fabric B was higher than that of fabric A; due to this, the HTP of fabric B was higher than that of fabric A. Among the fabrics C, D, and E, it was evident that changes in the HTP values of fabric D were minimal under different levels of moisture in both no air gap and 6 mm air gap conditions, whereas these values were maximized in the case of fabric E. This phenomenon could be explained based on the fiber content, fabric structure, and count of the fabrics. As fabric D is manufactured with synthetic aramid fibers, a ripstop structure, and comparatively low fabric count, it may not absorb much moisture; however, fabric E comprises natural cotton fibers with a twill structure and could absorb moisture [53]. Due to changes in the absorbed moisture level within the fabrics depending upon the fiber content, the HTP values could differ in both no air gap and 6 mm air gap conditions [54].

Furthermore, according to Table 2, THL values of the selected fabrics in no and 6 mm air gap conditions were all greater than 205 W/m^2^. As per the NFPA 1971 standard, the THL value of protective fabrics should not be less than 205 W/m^2^ to provide sufficient comfort to the wearers [55]. Based on this statement, it can be inferred that all the selected fabrics under different air gap conditions could provide sufficient thermal comfort to the wearers while wearing this fabric-based clothing. It has also been found that the HTP values of dry fabrics in the no air gap condition is different than the HTP values of dry fabrics in the 6 mm air gap condition. Based on the statistical analysis, it is evident that this difference is statistically significant with a *p*-value of 0.05. However, the 95% confidence interval (CI) of the difference in mean HTP values of dry fabrics in no air gap and air gap conditions varied from −9.67 to 0.01. As the CI values varies from + to −, it is not possible to conclude that this difference in HTP values is significantly higher or lower considering the current set of fabrics. However, the absolute negative CI value is much higher than the absolute positive CI value. Therefore, it can be concluded that the HTP values of the fabrics generally could increase in the presence of an air gap. This is because the air gap acts as an insulator that can trap significant amounts of dead air in between the fabrics and wearers’ skin [36]. As a result, the HTP values of the fabric could increase and that could help to provide better protection to the oilfield workers while exposed to heat [56]. 

According to Table 2, it is also evident that the THL values of fabrics A and B are nearly the same and are the lowest in both no air gap and 6 mm air gap conditions. This is because the air permeability of these fabrics is low, which might be due to the polymer coating that helps to transfer the metabolic heat and sweat vapor from the human body to the ambient environment [57]. As a result, the THL values of these fabrics are low. Nevertheless, fabric C has the maximum THL value. This could be because of the twill structure and synthetic fiber component in fabric C. Due to the twill structure, synthetic fibers, and comparatively high fabric count, moisture from the wearers’ skins could move to the outside of the fabric through wicking, and this situation would lower the thermal and evaporative resistance of the fabric. Eventually, the THL value of fabric C increases. Notably, fabric E also has a twill structure; however, it comprises natural cotton fibers. As a result, fabric E could absorb moisture and thereby increase the thermal and evaporative resistance of the fabric and THL would be higher. Notably, THL values of the fabrics were much lower in the presence of a 6 mm air gap in comparison to no air gap. Based on the statistical analysis, it is evident that this difference was significant with a *p*-value of <0.05. According to the 95% confidence interval test, this difference in upper level and lower levels of THL with a 6 mm air gap was always negative. This means the THL always gets lower in the presence of an air gap between the wearers’ skin and clothing. Actually, the trapped air within this gap acts as an insulator and resists the transmission of metabolic heat and sweat vapor from wearers’ bodies to the ambient environment. As a result, the thermal resistance and evaporative resistance becomes higher and that ultimately lowers the THL of the fabrics. 

### 3.1. Characterization to Identify Key Fabric Properties

**Figure 4:**
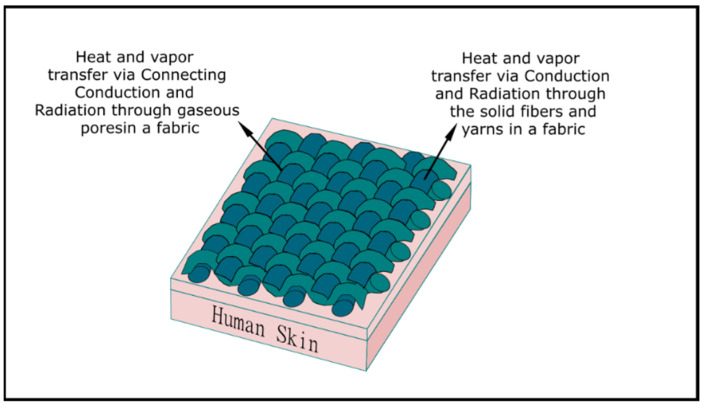
Heat and mass transfer through porous fabrics.

**Figure 5:**
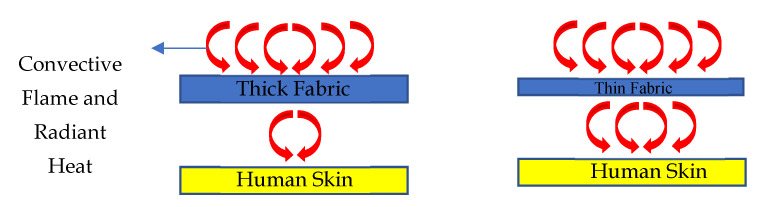
Heat transfer through thick and thin fabrics for HTP.

**Figure 6:**
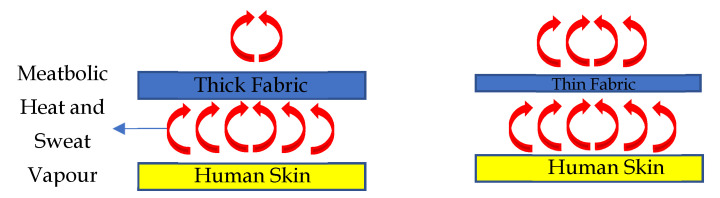
Heat transfer through thick and thin fabrics for THL.

Based on the linear regression analysis between the two fabric properties (thickness and air permeability) and performance (HTP and THL), it was found that the regression coefficient of thickness was the highest. This means that thickness is the key property that can affect the HTP/THL of dry and moistened fabric with and without an air gap. This is because the thickness of the fabric plays an important role in heat and mass transfer through the fabrics in the processes of convection, conduction, radiation, and/or mass diffusion [58]. In general, a fabric is a porous media with combined solid fibers and yarn phases as well as gaseous trapped air phases in between the fibers and yarns. Eventually, these solid (fiber and yarns) and gaseous (pores) phases could help to transfer the heat and vapor via convection, conduction, radiation, and/or mass diffusion [59] (Figure 4). In the presence of thick fabric, less convective flame and radiant heat transfer occurs through the fabrics’ solid and gaseous air phases in comparison to that of thin fabric; as a result, more time is required to generate burns on wearers’ skin and that can cause a higher HTP [58] (Figure 5). Similarly, thick fabric could transfer less metabolic heat and sweat vapor from human skin to the ambient environment through the solid and gaseous air phases in the fabric; as a result, the THL would be lower and generate more discomfort to wearers (Figure 6). As HTP and THL are phenomena associated with heat and mass transfer through the fabrics, thickness plays an important role for thermal protective and thermo-physiological comfort performance of fabrics. Therefore, thickness was used for the ANN modeling for predicting the thermal protective (HTP) and thermo-physiological comfort (THL) performance. In summary, it is notable that a fabric with high thickness could enhance the protective performance; however, it could significantly lower the comfort performance [52]. In this study, the main goal was to identify the key fabric properties that significantly affect performance, so thickness was chosen irrespective of its positive or negative effect on the performance.

### 3.2. ANN Modeling

**Figure 7:**
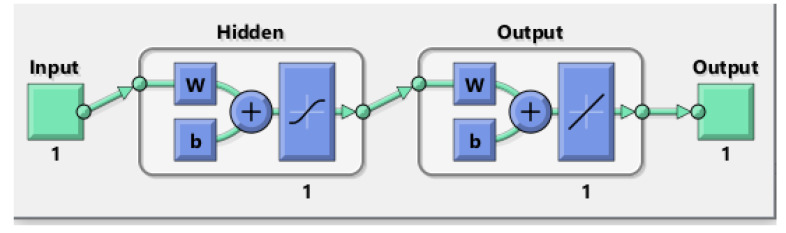
Diagram of the ANN model for HTP, where w represents the ANN weights and b represents bias; Input is the fabric property thickness and Output is HTP.

**Figure 8:**
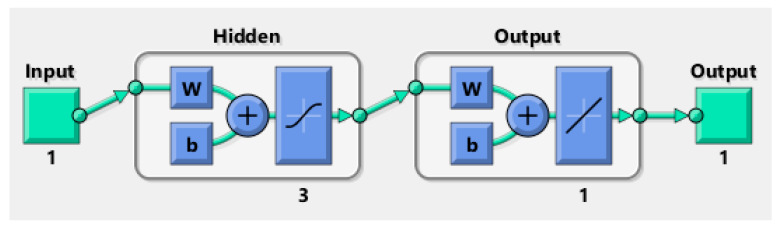
Diagram of the ANN model for THL, where w represents the ANN weights and b represents bias, Input is the fabric property thickness and Output is THL.

To predict the protective and comfort performance of moistened fabrics with a microclimate air gap using the key fabric properties identified in Section 3.1, the ANN approach was employed. The diagrams of these ANN models are presented in Figure 7 and Figure 8, where w represents the ANN weights and b represents the biases. The coding of these ANN models is presented in Appendix A and Appendix B. The best predictive ANN model for HTP had one neuron in its hidden layer (Figure 7), while the best predictive ANN model for THL had three neurons in its hidden layer (Figure 8).

The results of the ANN models for both thermal protective performance and thermo-physiological comfort performance are presented in Table 3. The Pearson correlation coefficient ‘r’ between the predicted HTP values from the ANN model and actual output HTP values was 0.54. This suggests a moderate-to-strong association between the predicted and actual HTP values. The Pearson correlation coefficient ‘r’ between the predicted THL values from the ANN model and actual output THL values was 0.33. This suggests a low-to-medium strength of association between the predicted and actual THL values. It is also evident that the RMSE of the ANN model for HTP was significantly (*p*-value < 0.05) lower than the RMSE of the ANN model for THL. This means that performance of the ANN model for HTP is higher than the performance of the ANN model for THL. Based on Pearson correlation coefficient and RMSE, it can be inferred that the ANN modeling approach could be successfully used to predict the thermal protective and thermo-physiological comfort performance of fabrics used in oilfield workers’ protective clothing. 

## 4. Conclusions

This study investigated the thermal protective and thermo-physiological comfort performance of fabrics used in oilfield workers’ protective clothing using an artificial neural network (ANN) modeling approach. For this, thermal protective and thermo-physiological comfort performance was characterized and the key fabric property affecting the performance, thickness, was identified. By employing the key fabric property, this study applied the ANN modeling approach to predict the thermal protective and thermo-physiological comfort performance of fabrics. This kind of ANN modeling approach could be used for conveniently predicting the performance of textile fabrics used in oilfield workers’ protective clothing. 

It is our expectation that user-friendly software-based models developed from this research will be used by procurement managers, designers, manufacturers, and/or researchers of oil and gas industry workers’ clothing to conveniently predict the performance of fabrics based on the fabrics’ physical properties. By using a limited number of fabrics, this study indicated that the ANN modeling approach could be useful for predicting the performance of fabrics used in oilfield workers’ clothing. In future, this model could be improved by considering a wide range of fabrics, moistures, and air gap conditions in order to accurately and realistically predict performance by using a neural network-based software application. Not only would this accomplishment have an enormous positive impact on the thermal protective textile sectors, buy it could also help to provide better occupational safety to oilfield workers from different hazards.

## Figures and Tables

**Table 1:** Selected fabrics and their properties.

Fabrics	Fiber Content	Fabric Structure			Fabric Properties				
			Fabric Count ^a^ (EPI × PPI; Total)	Weight ^b^ (Gram Per Square Meter)	Thickness ^c^ (mm)	Air Permeability ^d^ (cm^3^/cm^2^/s)	Thermal Resistance ^e^ (°C × m^2^/W)	Evaporative Resistance ^e^ (Pa × m^2^/W)	Absorbency Rating ^f^
A	50% Meta-aramid 50% Para-aramid	Twill	56 × 58; 114	237	0.37	6.94	0.016	6.3	100
B	100% Para-aramid	Twill	46 × 46; 92	237	0.41	6.2	0.014	7.47	100
C	50% Meta-aramid 50% Para-aramid	Twill	85 × 58; 143	272	0.48	13.58	0.007	2.59	0
D	50% Meta-aramid 50% Para-aramid	Ripstop	72 × 52; 124	204	0.49	33.42	0.021	4.03	50
E	60% Meta-aramid 40% Cotton	Twill	75 × 55; 130	237	0.47	47.84	0.021	2.45	0

EPI: Ends/Warps Per Inch; PPI: Picks/Wefts Per Inch. ^a^ Measured according to ASTM D3775; ^b^ Measured according to ASTM D3776; ^c^ Measured according to ASTM D1777; ^d^ Measured according to ASTM D737; ^e^ Measured according to ASTM F1868; ^f^ Measured according to AATCC 22.

**Table 2:** HTP and THL values of the selected fabrics.

Fabric	No Air Gap				6 mm Air Gap			
	HTP (cal/cm^2^) at Different Moisture Levels			THL (W/m^2^)	HTP (cal/cm^2^) at Different Moisture Levels			THL (W/m^2^)
	0%	20%	50%		0%	20%	50%	
A	5.97	No Heat Transfer (i.e., Very High HTP)	No Heat Transfer (i.e., Very High HTP)	542.79	13.14	No Heat Transfer (i.e., Very High HTP)	No Heat Transfer (i.e., Very High HTP)	237.8
B	7.01	No Heat Transfer (i.e., Very High HTP)	No Heat Transfer (i.e., Very High HTP)	511.79	15.47	No Heat Transfer (i.e., Very High HTP)	No Heat Transfer (i.e., Very High HTP)	229.5
C	7.48	11.62	10.16	797.09	8.42	13.07	17.39	371.7
D	6.39	6.08	6.9	628.68	13.71	13.88	13.96	269.87
E	7.61	11.50	15.32	764.79	7.87	12.73	18.52	266

**Table 3:** The Pearson correlation coefficient and RMSE of the developed ANN models.

Predicting Performance Parameters of Models	Thermal Protective Performance (HTP Value)	Thermo-Physiological Comfort Performance (THL Value)
Pearson correlation coefficient ‘r’	0.54	0.33
RMSE	4.37	293.03

## Appendix A. Coding for the ANN Model for HTP

% entering input data i.e., fabric property thickness x = [ 0.37 0.41 0.48 0.49 0.47 0.37 0.41 0.48 0.49 0.47 0.37 0.41 0.48 0.49 0.47 0.37 0.41 0.48 0.49 0.47 0.37 0.41 0.48 0.49 0.47 0.37 0.41 0.48 0.49 0.47]; % entering output data i.e., protective performance HTP t = [ 5.97 7.01 7.48 6.39 7.61 20 20 11.62 6.08 11.5 20 20 10.16 6.9 15.32 13.14 15.47 8.42 13.71 7.87 20 20 13.07 13.88 12.73 20 20 17.39 13.96 18.52]; % Choose a Training Function trainFcn = ‘trainlm’; % Levenberg-Marquardt backpropagation. ‘trainlm’ is usually fastest. % Create a Fitting Network hiddenLayerSize = 1;% number of hidden neurons. nethtp = fitnet (hiddenLayerSize,trainFcn); % Setup Division of Data for Training, Validation, Testing nethtp.divideParam.trainRatio = 70/100; nethtp.divideParam.valRatio = 15/100; nethtp.divideParam.testRatio = 15/100; nethtp.trainParam.epochs=3000;% number of training epochs. % Train the Network [nethtp,tr] = train (nethtp,x,t); % Test the Network y = nethtp (x); e = gsubtract (t,y); performance = perform (nethtp,t,y) % View the Network view (nethtp) % calculating the root mean square error rmse=sqrt (performance); % Plots figure, plotperform (tr) figure, plottrainstate (tr) figure, ploterrhist (e) figure, plotregression (t,y) % using the regression analysis to judge the network performance [m,b,r]=postreg (y,t); % saving the trained network save nethtp;

## Appendix B. Coding for the ANN Model for THL

% entering the input data i.e., thickness x = [ 0.37 0.41 0.48 0.49 0.47 0.37 0.41 0.48 0.49 0.47]; % entering the output data i.e., THL t = [542.79 511.79 797.09 628.68 764.79 237.8 229.5 371.7 269.87 266]; % Choose a Training Function trainFcn = ‘trainlm’; % Levenberg-Marquardt backpropagation. ‘trainlm’ is usually fastest. % Create a Fitting Network hiddenLayerSize = 3;% number of hidden neurons. netthl = fitnet (hiddenLayerSize,trainFcn); % Setup Division of Data for Training, Validation, Testing netthl.divideParam.trainRatio = 70/100; netthl.divideParam.valRatio = 15/100; netthl.divideParam.testRatio = 15/100; netthl.trainParam.epochs=3000;% number of training epochs. % Train the Network [netthl,tr] = train (netthl,x,t); % Test the Network y = netthl (x); e = gsubtract (t,y); performance = perform (netthl,t,y) % View the Network view (netthl) % Plots figure, plotperform (tr) figure, plottrainstate (tr) figure, ploterrhist (e) figure, plotregression (t,y) % using the regression analysis to judge the network performance [m,b,r]=postreg (y,t); % saving the trained network save netthl;
